# Experimental Study on the Compressive Strength and Fatigue Life of Cement Concrete under Temperature Differential Cycling

**DOI:** 10.3390/ma16237487

**Published:** 2023-12-02

**Authors:** Chengyun Tao, Lin Dong, Wenbo Fan, Tianlai Yu

**Affiliations:** 1 School of Civil and Transportation Engineering, Northeast Forestry University, Harbin 150040, China; taochengyun@nefu.edu.cn; 2 School of Civil and Architectural Engineering, Harbin University, Harbin 150076, China; dlin@hrbu.edu.cn (L.D.);

**Keywords:** cement concrete, temperature differential cycling, axial compressive strength, fatigue life

## Abstract

Concrete, as an engineering material with extremely wide applications, is widely used in various infrastructure projects such as bridges, highways, and large buildings. However, structures such as highways and bridges often need to be situated in variable and harsh service environments for long periods. They not only face cyclic reciprocating vehicle loads but also have to contend with the effects of temperature cycling. Therefore, studying the impact and mechanism of temperature differential cycling on the compressive strength and fatigue life of cement concrete has certain theoretical significance and practical value. This study employed a comprehensive experimental design to investigate cement concrete specimens subjected to typical temperature variations (20–60 °C) and different numbers of temperature differential cycling (0, 60, 120, 180, 240, 300). Axial compressive strength tests, ultrasonic tests, and compressive fatigue tests were conducted. The axial compressive strength test measured the compressive strength of the cement concrete. It was found that with an increase in the number of temperature differential cycling, the compressive strength exhibited a trend of an initial increase followed by a decrease: at 60 cycles, the strength increased by 10.8%, gradually declined; returned to near-initial strength at 120 cycles, and continued decreasing, reaching a decline of 19.4% at 300 cycles. The ultrasonic test measured the ultrasound velocity of the concrete specimens after different temperature differential cycling. It revealed a decreasing trend in ultrasound velocity with an increase in times of temperature differential cycling, showing a strong linear relationship between the ultrasound velocity loss and strength loss, confirming the correlation between the degree of concrete strength degradation and internal damage. The compressive fatigue test analyzed the fatigue life variation in cement concrete under different times of temperature differential cycling and stress levels, showing good adherence to the Weibull distribution pattern. Based on the approximation assumptions of log-normal distribution and the Weibull distribution, the Weibull distribution parameters for the compressive fatigue life of cement concrete under temperature differential cycling were obtained.

## 1. Introduction

Concrete is a solid stone-like material formed by mixing materials such as cement, aggregate, and water in suitable proportions. Because of its economy, high strength, and durability, concrete is extensively applied in large-scale projects and infrastructure such as high-rise buildings, bridges, tunnels, roads, and dams. For highway and other concrete structures, they must withstand the impact of changes in temperature while bearing the cyclic load of vehicle traffic. This is particularly true in regions of China like the Northeast and Northwest, where there are significant diurnal temperature variations, making it inevitable for concrete materials to experience long-term and repeated thermal effects [1,2]. It is well known that concrete has many components with different thermal properties. Uneven expansion and contraction between these components will occur due to continuous changes in temperature, which would thereby induce microcracks and compromise the structural integrity of the concrete [3], and then lead to degradation of concrete strength. At the same time, the cracks will continue to develop under the action of cyclic loads, and the resulting damage accumulation may accelerate the degradation process of the concrete to some extent, thus affecting the fatigue performance and durability of the concrete. Therefore, it is of vital necessity to study the effect of temperature differential cycling on the performance of concrete under static and cyclic loads.

Scholars at home and abroad have conducted in-depth studies on the mechanical properties of cement concrete under thermal effects. Li et al. [4] have researched the compressive strength, tensile strength, and modulus of elasticity of concrete after high temperatures and established a thermal-hygro-mechanical coupling mathematical model for concrete under high temperatures. Nadja [5] established a failure criterion formula for high-strength concrete after high temperatures through multiaxial strength tests and summarized the variation law of its uniaxial compressive strength with temperature. Ghandehari et al. [6] studied the strength loss rate and changing patterns of high-strength concrete after different high temperatures (100–600 °C) through axial tension tests, axial compression tests, and ultrasonic tests. The test results showed that the compressive and splitting tensile strengths of the concrete were both reduced, with a greater loss rate in splitting tensile strength. Wang et al. [7] analyzed the changes in strength and the variation law of ultrasonic wave speed of concrete after high temperatures and established a calculation formula for evaluating the strength damage and durability of concrete based on ultrasonic wave speed after different high temperatures. Gao [8] analyzed the performance changes in high-strength concrete after different high-temperature histories (100–900 °C) and established a relationship model between the fatigue damage degree of high-strength concrete and the high-temperature history. Paul Thanaraj et al. [9] conducted an experimental study on the mechanical properties and ultrasonic pulse velocity (UPV) of different concrete grades (M20–M50) subjected to varying durations of heating (15–240 min). The results indicate that the compressive strength, tensile strength, and flexural strength of concrete specimens ranging from M20 to M50 all suffered varying degrees of loss after heating. Additionally, UPV demonstrated a decreasing trend with prolonged heating durations. Kanagaraj et al. [10] investigated the effects of nanomaterials on the impact strength and microstructure of different grade concretes (M20–M50) at various high temperatures (250–1000 °C). The research findings revealed that irrespective of the type of nanomaterial additive in the concrete mixtures, the impact strength decreased as the temperature increased (from 250 to 1000 °C). Chen et al. [11] conducted experimental research on the compressive strength and freeze–thaw resistance of concrete under non-environmental conditions. The research results indicate that with an increase in the number of freeze–thaw cycles, the compressive strength of steam-cured concrete exhibits an overall decreasing trend, experiencing a strength loss ranging from 6.83 to 9.99%. Tang [12] conducted research on the compressive strength and peak strain of concrete incorporating different amounts of recycled aggregates and recycled rubber particles at various temperatures. The findings revealed that elevated temperatures significantly reduced the uniaxial compressive strength of recycled aggregate concrete. In summary, many scholars have conducted extensive research on the frost resistance, microscopic changes, or static mechanical properties (compressive strength, tensile strength, flexural strength, etc.) of concrete after being subjected to fixed temperature or temperature history. And all indicate that temperature changes have a significant impact on the mechanical properties of concrete. It is important to thoroughly study the impact of different temperature effects on the mechanical properties of concrete.

In recent years, in the study of temperature changes, the research on thermal fatigue damage of concrete has also attracted the attention of numerous scholars both domestically and internationally. An et al. [13] conducted a study on the mechanical properties and capillary water absorption of high-performance concrete under thermal cycling (20–65 °C), with results indicating that with an increase in the number of thermal cycles, the microstructure of the cement matrix, especially the interfacial transition zone, severely deteriorated, and the mechanical properties significantly degraded. Kanellopoulos et al. [14] carried out experimental research on the microstructure and mechanical properties of high-performance concrete subjected to thermal cycling (20 °C to 90 °C), revealing that after 30 thermal cycles, the mechanical properties of concrete improved; however, after 90 cycles, the porosity and crack width in the interfacial transition zone noticeably increased, leading to a significant decline in mechanical properties. Shokrieh et al. [15] performed experimental studies on the compressive strength and interfacial shear strength of polymer concrete under thermal cycling (25–30 °C, 25–70 °C, 30–70 °C), finding that the cycle from 25 to 70 °C had the most significant detrimental effect on the strength of the concrete. Xiao [16] studied the damage to concrete (C25, C40) after temperature differential cycling (20–40 °C, 20–50 °C, 20–60 °C), showing that with an increase in the number of cycles, the ultrasonic wave speed, relative compressive strength, and relative splitting tensile strength of the concrete tended to decrease without exception. Tang et al. [17] conducted experimental research on the effects of factors such as freeze–thaw cycles and concrete types on interfacial shear strength. The research findings indicate that when the strengths of conventional concrete and self-compacting concrete are relatively close, the freeze–thaw demonstrates a remarkable pronounced impact on the interfacial shear strength. In summary, many scholars have also paid attention to, and studied, the effect of thermal fatigue on the static load performance of concrete due to temperature changes. And it was found that thermal fatigue has varying degrees of influence on the strength and microstructure of concrete, which is worth further in-depth research.

The compressive fatigue performance of cement concrete has always been a hot topic in the field of civil engineering [18,19]. Since the early 20th century, a vast amount of research has been dedicated to gaining a deeper understanding of the fatigue behavior of concrete. Wu et al. [20] studied the compressive fatigue performance of high-strength concrete under cyclic load, providing empirical formulas for fatigue strength, strain, and so on. Wu et al. [20] also proposed a suggested formula for predicting fatigue failure of high-strength concrete based on static strain. Xue et al. [21] investigated the effect of freeze–thaw cycles on the compressive fatigue life of glue powder concrete, conducted reliability analysis on the distribution of fatigue life of concrete, and established a fatigue equation for glue powder concrete under the effect of freeze–thaw cycles based on the theory of equivalent damage. Byung [22] studied the impact of variable amplitude fatigue loads on the fatigue life of concrete beams, finding that the fatigue life of concrete conformed to the two-parameter Weibull distribution, and that the shape and scale parameters in the Weibull model were not identical at different stress levels. Ou et al. [23] researched the impact of freeze–thaw cycles on the net bending strength, fatigue strength, and fatigue life of concrete beams, discovering that the bending fatigue life of concrete followed the Weibull distribution, and that the reliability probability of its fatigue life decreased with the increase in the number of freeze–thaw cycles. It can be found that research on the fatigue performance of concrete mostly focuses on the effects of freeze–thaw cycles or mechanical loads. And research has shown that the fatigue life of concrete is significantly influenced by various factors such as freeze–thaw cycles. In fact, freeze–thaw cycles are also a type of temperature differential cycling with a large range of changes that can cover the solidification point of water in concrete. However, further in-depth research is also needed to pay attention to the temperature differential cycling effect that has not reached the solidification point of water in concrete with a small range of changes.

In summary, there is in-depth research on the static mechanical properties of concrete under fixed high or low temperatures, freeze–thaw or temperature cycling conditions (such as compressive and tensile strength, damage patterns, etc.), or in other complex environments [24,25,26]. However, the research on the fatigue performance of concrete predominantly focuses on single-factor fatigue effects, such as load fatigue or thermal fatigue, with limited studies on the combined effects of both. In fact, load fatigue and thermal fatigue often coexist: the road concrete not only endures the effect of fatigue loading caused by the cyclic reciprocation of vehicles and other loads and the seasonal effects of freeze–thaw cycles, but also faces the periodic cyclic temperature differentials caused by factors such as sunlight and radiation, which would not change the state of water in the concrete in actual engineering environments. Particularly in regions of Northeast and Northwest China, significant day–night temperature differences expose highway concrete to repeated cycles of temperature variation. Therefore, to further align with actual engineering and enhance the application safety of concrete in projects, it is of great theoretical and practical significance to deeply study the impact of temperature differential cycles on the compressive strength and fatigue life of cement concrete.

To assess the impact of temperature differential cycling on the compressive strength, fatigue performance, and extent of damage to cement concrete more comprehensively and systematically, this study conducts axial compression tests, ultrasonic tests, and compressive fatigue tests at different stress levels on cement concrete specimens that have undergone different temperature differential cycling. It explores the patterns of change in compressive strength and the loss of ultrasonic wave speed, and also conducts statistical analysis of their fatigue life based on the two-parameter Weibull distribution model, in order to provide scientific theoretical basis and technical support for the application and optimization of concrete.

## 2. Materials and Methods

### 2.1. Experimental Materials and Specimens

The cement used in the experiment is ordinary Portland cement of P.O 42.5 grade of Jidong brand (Cangzhou, Hebei Province, China), which is widely used in various concrete products and building structures due to its superiority of early strength and durability. The main physical and mechanical properties are shown in Table 1.

The raw materials for the cement concrete in this experiment primarily consist of P.O 42.5 ordinary Portland cement, Class II fly ash, S95 slag powder, medium sand, and crushed stone (granite crushed stone, 5–31.5 continuous grading), SM-1 type high-performance pumping agent, and water. The concrete mix proportion is designed referring to the standard JGJ55-2011 “Specification for mix proportion design of ordinary concrete” [27] with a design strength grade of C55 and a slump of 200 mm. The specific mix proportions are shown in Table 2.

The experiment follows the standards of GB/T 50081-2019 “Standard for test methods of concrete physical and mechanical properties” [28] and GB/T 50082-2009 “Standard for Test Methods of Long-term Performance and Durability of Ordinary Concrete” [29], to determine the size and quantity of concrete specimens. To better align with practical engineering applications, prism specimens sized at 100 × 100 × 300 mm were selected. A total of 12 specimens were established for each temperature differential cycling count (0, 60, 120, 180, 240, 300). Among these, 3 specimens were allocated for conducting axial compressive strength tests to measure the compressive strength along the specimen’s axis, denoted as *f*_r_. Additionally, 9 specimens were designated for axial compressive fatigue tests, distributing 3 specimens for each stress level (S = 0.70, S = 0.80, S = 0.90). The dimensions and quantity of the concrete specimens are detailed in Table 3.

The concrete specimens were demolded 24 h after fabrication and cured under standard conditions (temperature of 20 ± 2 °C, RH > 90%) for 28 days before the relevant tests were conducted.

### 2.2. Equipment and Methods of Experiment

#### 2.2.1. Temperature Differential Cycling Test

**Figure 1:**
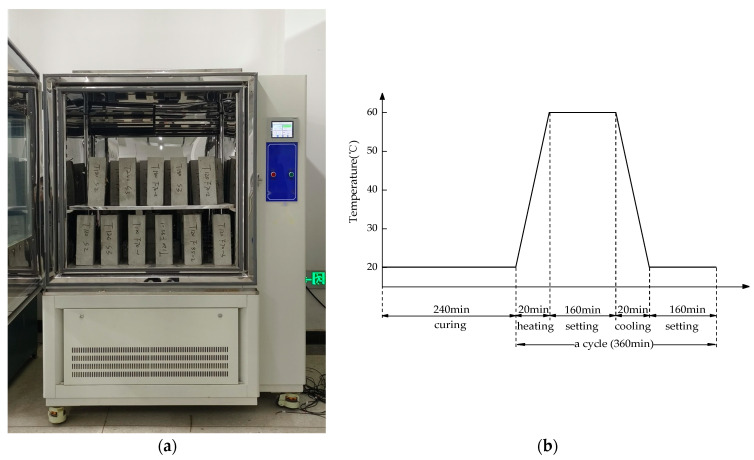
The equipment and mechanism of temperature differential cycling. (**a**) high–low temperature alternating test chamber. (**b**) mechanism of temperature differential cycling.

The equipment used for temperature differential cycling test was the high–low temperature alternating test chamber from Shanghai Yihua Instrument Equipment Corporation (Shanghai, China) (as shown in Figure 1a). The concrete specimens were placed in the temperature control chamber, with the lowest and highest temperatures of boundary set to 20 °C and 60 °C, respectively. The heating and cooling rates of the temperature control chamber were set at 2 °C/min to conduct the temperature differential cycling. The number of temperature differential cycling in this test were set at 0, 60, 120, 180, 240, and 300 times, respectively, after which related tests and studies were conducted. For each count of temperature differential cycling, 15 specimens were extracted from the environmental chamber for testing purposes. Among these, 3 were designated for axial compressive strength tests, 9 were allocated for axial compressive fatigue tests, and the remaining 3 were utilized for ultrasonic testing (which is a non-destructive test). The specimens used for ultrasonic testing served as reference samples and, after the test, were returned to the environmental chamber to continue undergoing temperature differential cycling. It is necessary to consider that during the changes in temperature, there may be a certain temperature differential between the surface and interior of the concrete specimens, and the existence of that may lead to an increase in thermal stress inside the specimen, thus exacerbating the deterioration of strength. To eliminate the effects caused by the temperature differential between the surface and interior of the specimen, tests were conducted prior to the thermal differential cycling tests. It was found that for the concrete specimen when the temperature control chamber just reached 60 °C, placing it in the temperature control chamber for 160 min could allow the center temperature of the specimen to rise to 60 °C and, similarly, for the concrete specimen when the temperature control chamber just reached 20 °C, placing it in the temperature control chamber for 160 min could allow the center temperature of the specimen to drop to 20 °C. Therefore, the mechanism of temperature differential cycling established in this study is as follows:

(I) Place the specimen at the starting temperature (20 °C) for 240 min;
(II) Heat the temperature control chamber up at a rate of 2 °C/min to the highest boundary temperature (60 °C) and then maintain a constant temperature for 160 min, with a total duration of 180 min for this process;
(III) Cool the temperature control chamber down at a rate of 2 °C/min to the lowest boundary temperature (20 °C) and then maintain a constant temperature for 160 min, with a total duration of 180 min for this process;
(IV) Repeat steps (II) and (III) to complete a temperature differential cycling.

To ensure the accuracy of the test, the concrete specimens are first placed at the starting temperature (20 °C) for 240 min, and once the temperature of the concrete block stabilizes, the temperature differential cycling treatment is started. The total duration of a temperature differential cycling lasts 360 min. The schematic diagram of the mechanism of temperature differential cycling is as shown in Figure 1b.

#### 2.2.2. Axial Compressive Strength Test

The axial compression strength test was conducted using a TYA-3000E type microcomputer-controlled constant loading pressure testing machine manufactured by Wuxi Xinluda Instrument Equipment Corporation (Wuxi, China). The procedures of the test followed the GB/T 50081-2019 “Standard for test methods of concrete physical and mechanical properties” [28]. For each count OF temperature differential cycling (0, 60, 120, 180, 240, 300), 3 specimens were selected, resulting in a total of 18 specimens:

(I) Specimens of dimensions 100 mm × 100 mm × 300 mm were chosen as prism specimens. Inspection was carried out to verify the specimen dimensions and shape, ensuring that the dimensional tolerances met the specifications.
(II) The surfaces of the upper and lower compression plates of the testing machine, as well as the surfaces of the specimens, were wiped clean. The specimens were then positioned vertically on the lower compression plate, aligning the specimen axis with the center of the lower compression plate.
(III) The testing machine was activated, and the loading during the test was continuous and uniform, with a loading rate set as 0.5–0.8 MPa/s.
(IV) The failure load was recorded during the test process.

#### 2.2.3. Axial Compression Fatigue Test

**Figure 2:**
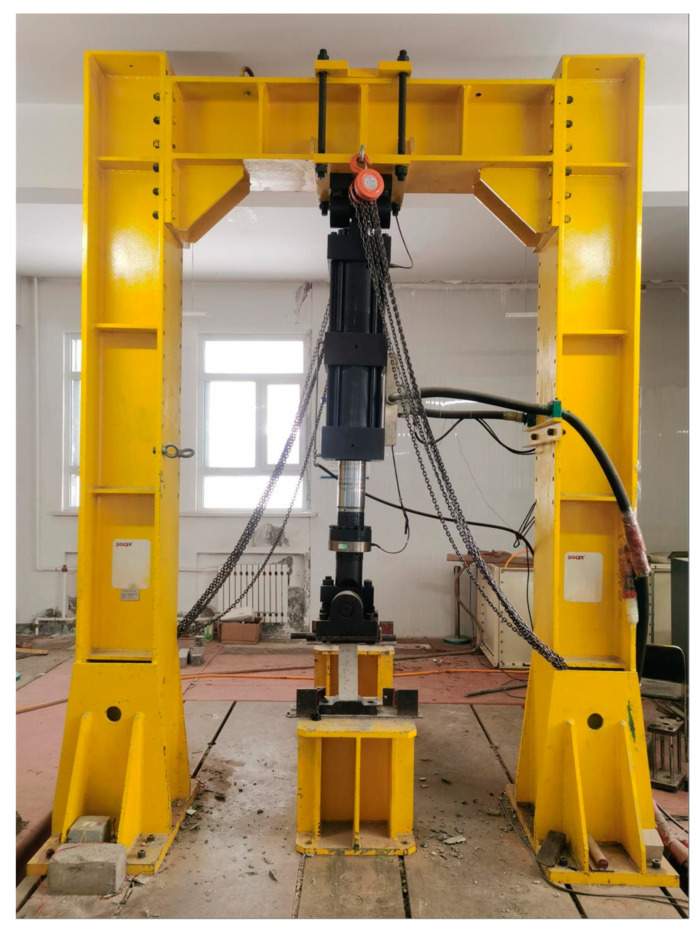
PLS-500 dynamic and static fatigue testing machine.

The equipment for the axial compressive fatigue test was the PLS-500 dynamic and static fatigue testing machine from the Jinan East Testing Machine Factory (Jinan, China) (shown in Figure 2). The testing procedure adhered to the guidelines specified in GB/T 50082-2009 “Standard for test methods of long-term performance and durability of ordinary concrete” [29]. For each count of temperature differential cycling (0, 60, 120, 180, 240, 300), 9 specimens were selected, resulting in a total of 54 specimens. Among these, for each stress level (S = 0.70, S = 0.80, S = 0.90), 3 specimens were allocated:

(I) The compressive strength of the specimens obtained from the previous axial compressive strength test was recorded.
(II) Static compression deformation was performed on the fatigue testing machine for centering: initial centering was conducted at 20% of *f*_r_, followed by a second centering at 40% of *f*_r_. The position of the specimens was adjusted until the difference in deformation on both sides of the specimen was less than 5% of the average value.
(III) The fatigue testing machine was then activated. A sine wave was chosen as the loading form, with a loading frequency set to 4 Hz; the magnitude of the fatigue test loading was determined based on the stress ratio *Q*, stress level *S*, and the axial compressive strength *f*_r_: the stress ratio set to *Q* = 0.1 (where *Q* is the ratio of the minimum to maximum fatigue stress, *Q* = *f*_min_/*f*_max_).Three stress levels were selected in this study: *S*_1_ = 0.70, *S*_2_ = 0.80, *S*_3_ = 0.90 (where *S* is the ratio of the maximum fatigue stress to the static compressive strength, *S* = *f*_max_/*f*_r_).
(IV) The number of loading cycles N from the beginning of loading to complete failure was recorded as the fatigue life of the specimen. Moreover, to improve the efficiency of the test, the number of loading cycles *n* = 2,000,000 was set as the test termination threshold in this study. That is: if the specimen completely fails when *n* < 2,000,000 cycles, the test is terminated, and the loading cycles at this point are recorded as the fatigue life of the specimen (N = *n*); if the specimen has still not completely failed when *n* = 2,000,000 cycles, the test is also terminated, and the residual strength of the specimen is measured at this time.

#### 2.2.4. Ultrasonic Test

**Figure 3:**
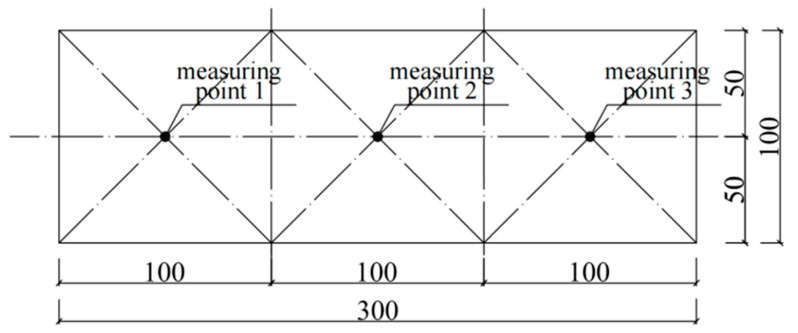
Layout of measuring points for ultrasonic test.

The ultrasonic test was carried out to measure the acoustic time using the HC-U81 concrete ultrasonic detector. The procedures of the test followed GECS21:2000 “Technical Specification for Inspection of Concrete Defects by Ultrasonic Method” [30]. Three specimens were designated for the acoustic time measurement. As ultrasonic testing is non-destructive, the specimens were returned to the temperature control chamber to continue undergoing temperature differential cycling after the measurement. To investigate the damage to the cement concrete under temperature differential cycling more accurately, an ultrasonic test for the specimens was designed after every 30 thermal differential cycles in this study. The steps for conducting the acoustic time measurement in the ultrasonic test were as follows:

(I) Use the cross-measurement method to arrange the measuring points, marking ultrasonic wave transmitting and receiving points on two parallel faces of the specimens, as depicted in Figure 3.
(II) Couple the transmitting transducer and receiving transducer to the corresponding measuring points and initiate the instrument to transmit ultrasonic waves.
(III) Record the transit time value *t* (μs).
(IV) Record ultrasound velocity: Given the specimen dimensions (100 × 100 × 300), for a measuring distance *l* = 100 mm, the sound velocity v = *l*/*t* (km/s) can be calculated. Perform 3 measurements at each measuring point and take the average value as the sound velocity for that measuring point.

### 2.3. Procedure of Experiment

Due to the large number of concrete specimen indicators measured in this study, as well as the time-consuming nature of the designed thermal differential cycle and fatigue tests, it is necessary to design a rigorous plan before conducting the experiments to improve test efficiency. To better simulate real-world engineering conditions, this study selected a temperature differential range of 20–60 °C. The relatively minor temperature variations and the influence on the mechanical properties of the specimens are also limited. Based on existing research, we believe that subjecting concrete specimens to mechanical performance tests after every 60 cycles of temperature differential changes adequately reflects the pattern of concrete’s mechanical properties in response to temperature variations. However, in terms of ultrasonic testing, as internal concrete damage cannot be observed through visual inspection and considering that ultrasonic testing is a non-destructive method, for a more accurate investigation into the development pattern of internal concrete damage due to temperature differential cycling, we chose to conduct an ultrasonic test on the specimens after every 30 cycles of temperature differential cycling.

**Figure 4:**
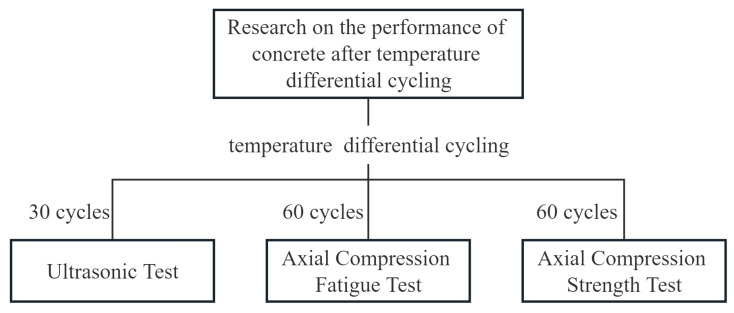
Flow chart of the experiments.

The procedure of the experiments carried out in this study is shown in Figure 4.

## 3. Results and Analysis of Experiment 

### 3.1. The Influence of Temperature Differential Cycling on Compressive Strength of Concrete 

**Figure 5:**
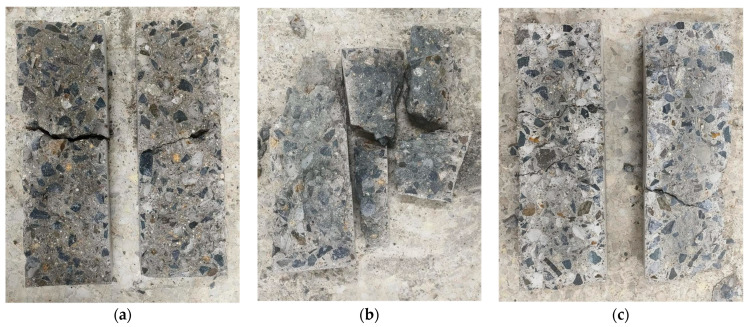
Fracture surface of the specimens after temperature differential cycling. (**a**) un-cycled; (**b**) 120 cycles; (**c**) 300 cycles.

By observing the changes in color in the fracture surfaces of the cement concrete specimens, it can be found that the color changed gradually from the dark gray of the un-cycled specimens to the lighter gray after 120 cycles, and then to gray–white after 300 cycles (as shown in Figure 5).

**Figure 6:**
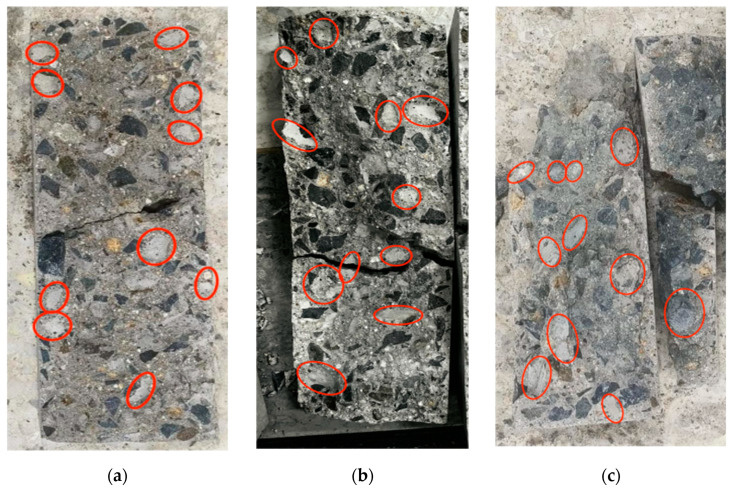

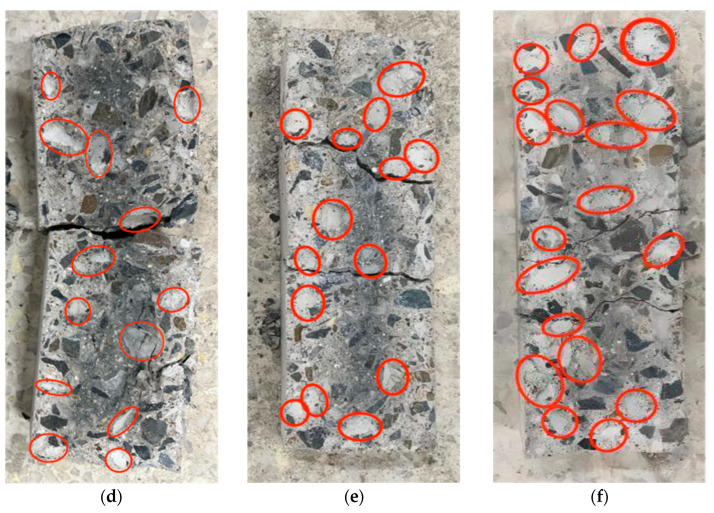
The failure surfaces of specimens under uniaxial compressive stress after temperature differential cycling. (**a**) un-cycled; (**b**) 60 cycles; (**c**) 120 cycles; (**d**) 180 cycles; (**e**) 240 cycles; (**f**) 300 cycles. Where, the red circles represent the fracture occurrences at the interface between the coarse aggregate and cement paste.

To analyze and discuss the impact of temperature differential cycling on the performance of concrete specimens further, the experimental research on the compressive performance of concrete was also conducted in this study as well as observing the apparent characteristics of the specimens after temperature differential cycling. The failure surfaces of the cement concrete specimens under uniaxial compressive stress after temperature differential cycling are shown in Figure 6.

It was observed that after a fewer number of temperature differential cycling (60 and 120 cycles), there were no obvious through cracks in the concrete specimens before loading. However, when the load reached a certain level, cracks rapidly developed and extended in a short time until they penetrated the entire specimen. After a greater number of thermal cycles (180, 240, and 300 cycles), cracks could be observed in the early stages of loading, and the specimens showed a denser pattern of cracks at the center.

Furthermore, it can be seen from the morphology of the fracture surface in Figure 6 that without temperature differential cycling, there were 10 fracture occurrences at the interface between the coarse aggregate and cement paste with a maximum diameter of 21.2 mm; after 120 thermal cycles, there were 11 occurrences with a maximum diameter of 25.5 mm; and after 300 thermal cycles, there were 18 occurrences with a maximum diameter of 28.3 mm.

This indicates that with the continuous progression of temperature differential cycling, the number of damaged points on the interface between coarse aggregates and cement paste increases gradually. This phenomenon may stem from the differing thermal effects between coarse aggregates and the cementitious matrix when subjected to temperature fluctuations, leading to distinct deformations between them. These deformation disparities could trigger the formation of microcracks in the transitional area of the interface between coarse aggregates and cement paste. These microcracks continue to propagate and extend under the influence of temperature cycling, progressively compromising the overall integrity of the interface transitional zone and deteriorating its performance. Additionally, we have observed that with the ongoing temperature differential cycles, the points of damage gradually extend from the edge region of the specimen towards the central area. This suggests that the impact of temperature cycling on the specimen’s surface is more significant than its internal regions.

The failure loads and compressive strengths of the cement concrete specimens after different temperature differential cyclin measured by the axial compression tests are shown in Table 4. It is worth mentioning that, according to GB/T 50081-2019 “Standard for Test Method of Mechanical Properties on Ordinary Concrete” [28], the compressive strength of the non-standard specimens used in this study (100 mm × 100 mm × 300 mm) must be converted using Formula (1).

(1) $$f_{\mathrm{cp}}=af_{t}$$

where, a is the size conversion coefficient, a = 0.95; *f*_t_ is the compressive strength of the specimen obtained from the test.

It can be found from Table 4 that comparing the specimens at the reference temperature (20 °C), those subjected to temperature differential cycling initially exhibit an increase in compressive strength. However, after 60 cycles of temperature variation, the compressive strength begins to decrease. Furthermore, as the number of temperature differential cycling increases, the reduction in compressive strength of the cement concrete specimens becomes more pronounced. That is, for cement concrete, the compressive strength shows an overall trend of “first increasing and then decreasing” during the process of temperature differential cycling.

**Figure 7:**
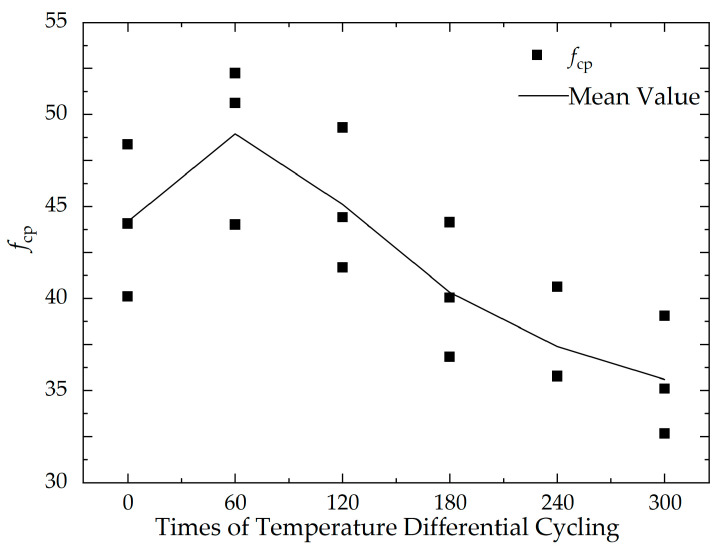
Axial compressive strength of specimens after temperature differential cycling.

To observe the impact of temperature differential cycling on the strength of cement concrete more intuitively, a graph has been plotted to show the change in the average compressive strength of each group of specimens with the number of temperature differential cycling, as shown in Figure 7. The slopes and intercepts of each segment of the curve can be found in Table 5.

It can be seen from Figure 7 and Table 5 that the strength of the concrete specimens improved after the initial 60 cycles, but when reaching 120 cycles, the strength falls back to near the compressive strength values at the baseline temperature, and it continues to decline with an increasing number of temperature cycles. On one hand, due to the different thermal effects among the various components of the concrete specimens, the size and rate of thermal deformation varies during the changes in temperature, leading to the formation of microcracks between components, thus affecting the integrity and degrading the strength of the specimens. On the other hand, considering the actual experimental conditions, the specimens in the temperature control chamber experience a temperature difference between their surface and interior during the changes in the temperature control chamber, leading to thermal stress and promoting the formation of microcracks, further degrading the strength of the concrete specimens.

**Figure 8:**
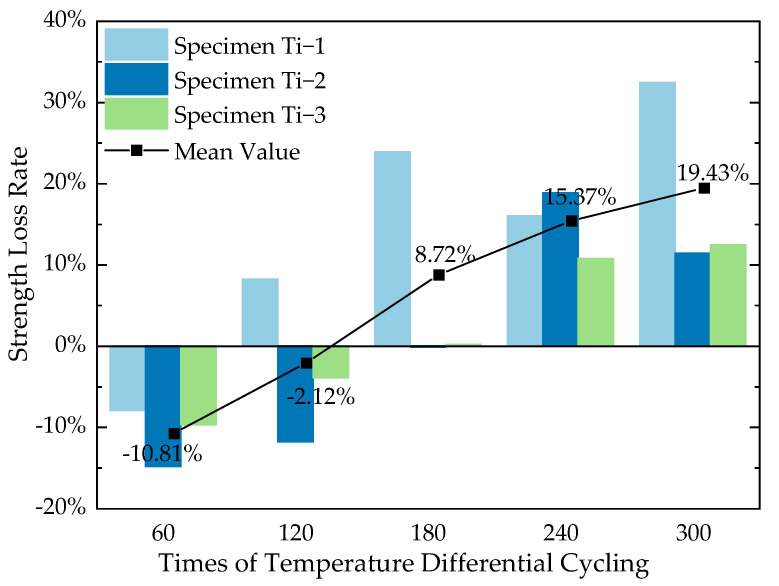
Strength loss rate of cement concrete specimen after temperature differential cycling.

The strength loss rate *R*_s_ (Equation (2)) was selected in this study to characterize the strength degradation of cement concrete specimens after different temperature differential cycling quantitatively. The strength loss rate of the concrete specimens obtained from the axial compression test is shown in Table 6, and a graph depicting the average compressive strength loss rate as a function of the number of temperature cycles is presented in Figure 8.

(2) $$R_{s}=\left(f_{\mathrm{cp}}^{T}-f_{\mathrm{cp}}\right)/f_{\mathrm{cp}}\times 100\%$$

where *f*_cp_^T^ is the compressive strength of the concrete specimens after different temperature differential cycling, and *f*_cp_ is the compressive strength of the concrete specimens at the baseline temperature (20 °C).

It is observed that comparing concrete that has not undergone temperature differential cycling, the strength loss of concrete specimens shows negative values in the early stages of cycling, indicating an upward trend in strength. However, after 180 cycles, some specimens begin to show a significant decrease in strength, by more than 20%. After 240 cycles, the loss in compressive strength of the specimens reaches 10–20%, and after 240 cycles, the compressive strength loss even reaches 10–30%.

By observing the curve of the average compressive strength loss rate of concrete specimens in Figure 8, it can be found that in the first 120 cycles, the strength loss rate of concrete specimens shows as a negative value, meaning the strength of the specimens has generally increased compared to that at the baseline temperature, and it reaches its maximum value at the 60 cycles. During 120–180 cycles, the strength loss rate gradually increases to a positive value, indicating that the strength of the specimens gradually decreases to below that at the baseline temperature and continues to diminish. At the same time, it can be found from the rate of the curve that the increase in the strength loss rate is gradual relatively during 60–120 cycles; and grows significantly during 120–180 cycles; then returns to a gradual pace during 180–300 cycles.

The possible reasons are as follows: in the early stages of temperature differential cycling (first 120 cycles), the rise in temperature promotes the hydration of some cement particles that have not yet fully hydrated, which could enhance the internal density of the concrete and thus increase the strength of the concrete specimens. Within the first 60 cycles, the strength increase caused by the hydration of the cement is greater than the deterioration caused by the temperature differential cycling on the strength of the concrete specimens, hence showing an increase in strength. During 120–180 cycles, as the number of temperature differential cycling increases, the unhydrated cement and fly ash particles decrease gradually, the hydration process slows down, the enhancement effect on strength weakens, and the impact of temperature cycling becomes the dominant factor, thus showing a strength loss rate rising from a negative to a positive value, with a relatively large rate of increase. In the later stages of temperature cycling (180–300 cycles), there are some microcracks inside the concrete specimens already, and the existence of those weakens the thermal stress caused by the different thermal properties between the concrete components, thus having a smaller impact on strength, which is reflected in the slowing rate of increase in the strength loss rate of the concrete specimens.

### 3.2. Effect of Temperature Differential Cycling on the Fatigue Performance of Concrete

The fatigue life of concrete specimens subjected to different temperature differential cycling under various stress levels is measured by axial compression fatigue tests as shown in Table 7.

The fatigue life of concrete, which means the maximum number of load cycles a concrete can withstand before failure under cyclic loading conditions, is one of the important characteristics for evaluating the service performance of concrete. In fact, the fatigue life of concrete is closely related to its own heterogeneity, the number of temperature differential cycling, and the level of the load, and thus typically exhibits a large degree of variability. Therefore, it is necessary to introduce probabilistic statistical methods to describe the distribution of fatigue life of concrete more accurately.

It is well known that the distribution of fatigue life of concrete usually follows both a two-parameter Weibull distribution and a log-normal distribution. The two-parameter Weibull distribution is a continuous distribution that describes the distribution of fatigue life of materials through two parameters: shape and scale. Due to its convenience and straightforward derivation, it is widely used in the statistical analysis of material life [31,32,33,34]. However, the two-parameter Weibull distribution model often cannot avoid the use of mathematical methods such as the maximum likelihood method or the moment estimation method, which involve multiple dimensions of equations and large amounts of computation, thus limiting its practical application. Therefore, based on the premise that the concrete fatigue life conforms to both the log-normal distribution and the two-parameter Weibull distribution, the assumption of the log-normal distribution’s approximation was used in this study to estimate the distribution parameters of the two-parameter Weibull distribution to improve calculation efficiency.

Assume the target variable *x* follows a two-parameter Weibull distribution, the density function of which can be describe as [35]:

(3) $$f_{w}(x)=\left(\frac{\beta }{\eta }\right){\left(\frac{x}{\eta }\right)}^{\beta -1}\exp\left\{-{\left(\frac{x}{\eta }\right)}^{\beta }\right\}\;x>0,\;\beta >0,\;\eta >0$$

where *β* is the shape parameter; *η* is the scale parameter.

Assume the target variable *x* follows a log-normal distribution at the same time, the density function of which can be describe as [36]:

(4) $$f_{\ln}(x)=\frac{1}{\sqrt{2\pi \sigma }x}\exp\left[-\frac{{\left(\ln x-EX\right)}^{2}}{2\sigma^{2}}\right]\;x>0$$

where *EX* is the logarithmic mean of the sample variables, and *σ*^2^ is the variance between the sample variables.

Then, the characteristic functions of the two distribution functions are:

(5) $$P_{w}\left(t\right)=\int_{x>0}e^{itx}f_{w}(x)dx$$

(6) $$P_{\ln}\left(t\right)=\int_{x>0}e^{itx}f_{\ln}(x)dx$$

Performing a Taylor expansion on the characteristic functions:

(7) $$P_{w}\left(t\right)=\sum_{k=1}^{n-1}P_{w}^{\left(k\right)}\left(0\right)t^{k}/k!+O_{1}\left(t^{n}\right)$$

(8) $$P_{\ln}\left(t\right)=\sum_{k=1}^{n-1}P_{\ln}^{\left(k\right)}\left(0\right)t^{k}/k!+O_{2}\left(t^{n}\right)$$

According to the continuity theorem, when the two functions are approximate, it holds that:

(9) $$\underset{n\to \infty }{\lim}P_{w}\left(t\right)=\underset{n\to \infty }{\lim}P_{\ln}\left(t\right)$$

By combining Equations (7)–(9) and approximating the first three terms of the Taylor expansion, it can be obtained that:

(10) $$\int_{x>0}x^{n}f_{w}(x)dx=\int_{x>0}x^{n}f_{\ln}(x)dx\;n=0,1,2$$

For *n* = 0, the left and right sides of Equation (10) are equal;

For *n* = 1, 2, there are additional equations derived:

(11) $$E_{w}\left(x\right)=E_{\ln}\left(x\right)\;n=0$$

(12) $$\int_{x>0}{\left[x-E_{w}\left(x\right)\right]}^{2}f_{w}(x)dx=\int_{x>0}{\left[x-E_{\ln}\left(x\right)\right]}^{2}f_{\ln}(x)dx\;n=2$$

where *E*(·) is the expected value of the sample.

It can be obtained from Equations (3), (4), (11) and (12):

(13) $$\eta \Gamma \left(1+1/\beta \right)=\exp\left(EX+\sigma^{2}/2\right)$$

(14) $$\eta^{2}\left[\Gamma \left(1+2/\beta \right)-\Gamma^{2}\left(1+1/\beta \right)\right]=\left[\exp\left(2EX+\sigma^{2}\right)\right]\left[\exp\left(\sigma^{2}\right)-1\right]$$

where Γ(·)is the gamma function.

For ease of calculation, let:

(15) $$\omega \left(\beta \right)=\Gamma \left(1+1/\beta \right)/\sqrt{\left[\Gamma \left(1+2/\beta \right)-\Gamma^{2}\left(1+1/\beta \right)\right]}$$

Performing calculations of ω(*β*) within the range *β =* [1, 3.175], we can obtain a corresponding table as shown in Table 8.

According to Equation (15), Equations (13) and (14) can be further simplified:

(16) $$\omega \left(\beta \right)=1/\sqrt{\exp\left(\sigma^{2}\right)-1}$$

(17) $$\eta =\exp\left(EX\right)\sqrt{\exp\left(\sigma^{2}\right)}/\Gamma \left(1+1/\beta \right)$$

Thus, we can calculate the value of ω(*β*) using Equation (16), then refer to Table 8 to find the shape parameter *β*, and obtain the scale parameter *η* using Equation (17), thereby achieving the estimation of the two-parameter Weibull distribution parameters through the assumption of the log-normal distribution’s approximation. The calculated values of the Weibull distribution parameters for the compressive fatigue life of the cement concrete in this study are shown in Table 9.

The Kolmogorov–Smirnov (K–S) test method was used in this study to evaluate the goodness of fit between the experimental distribution of the fatigue life of cement concrete and the two-parameter Weibull distribution model determined by the calculated distribution parameters [37,38]. As one of the most commonly used non-parametric methods, the essence of the K–S test method’s evaluation of the goodness of fit is to describe the distance between the empirical function of the sample distribution and the distribution function of the reference distribution quantitatively. The size of the distance reflects the goodness of fit between the sample and the reference distribution.

Let *F*_n_(*x*) be the empirical distribution function of the sample:

(18) $$F_{n}\left(x_{i}\right)=i/n$$

where n is the sample size, and *i* is the sample index.

Let *P*_n_(*x*) be the cumulative probability distribution function of the reference distribution, then calculate the distance *D_i_* between the two distributions:

(19) $$D_{i}=\left|F_{n}\left(x_{i}\right)-P_{n}\left(x_{i}\right)\right|$$

According to the significance of the K–S test, the maximum deviation between *F*_n_(*x*) and *P*_n_(*x*), denoted as *D*_max_, is the statistic of K–S test:

(20) $$D_{\max}=\underset{0\leq i\leq n}{\max}D_{i}=\underset{0\leq i\leq n}{\max}\left|F_{n}\left(x_{i}\right)-P_{n}\left(x_{i}\right)\right|$$

For this study, the empirical cumulative probability distribution function *F*_n_(*x*) corresponds to the cumulative probability distribution function of the compressive fatigue life of the cement concrete obtained from the experiment, and *P*_n_(*x*) corresponds to the cumulative probability distribution function of the two-parameter Weibull model:

(21) $$P_{n}\left(x_{i}\right)=1-\exp\left(-\frac{x_{i}{}^{\beta }}{EX^{\beta }}\right)$$

Taking the distribution of compressive fatigue life of cement concrete specimens under stress level S = 0.70 without temperature differential cycling (T = 0) as an example, the K–S test for the goodness of fit of the two-parameter Weibull distribution is shown in Table 10.

It can be seen that the value of test statistic calculated out is *D*_max_ = 0.3656, and the standard value of which, according to the critical value table of the K–S test standard, is *D*_C_ = 0.734 (at the significance level of 5%, on 3 test repetitions) [25]. Since *D*_max_ < *D*_C_, the goodness of fit between the distribution of fatigue life and the two-parameter Weibull distribution can pass the K–S test, meaning that the distribution of compressive fatigue life of cement concrete under the condition of S = 0.70, T = 0 conforms to the two-parameter Weibull distribution. Using this method, the goodness of fit between the fatigue life distribution of cement concrete under all working conditions and the two-parameter Weibull distribution is tested, the results are shown in Table 11.

It can be seen that for all stress levels and temperature cycling conditions in this study, the goodness of fit of the two-parameter Weibull distribution of concrete specimen fatigue life can pass the K–S test, which means that at a 5% significance level, the fatigue life distribution of cement concrete can be well described by the two-parameter Weibull distribution.

To characterize the impact of temperature cycling frequency on concrete life quantitatively, an exponential function was applied in this study to fit the variation in fatigue life with temperature cycling frequency, as shown in Equation (22):

(22) $$N=a\exp\left(bT+c\right)$$

where a, b, c are coefficient constants related to stress levels.

**Figure 9:**
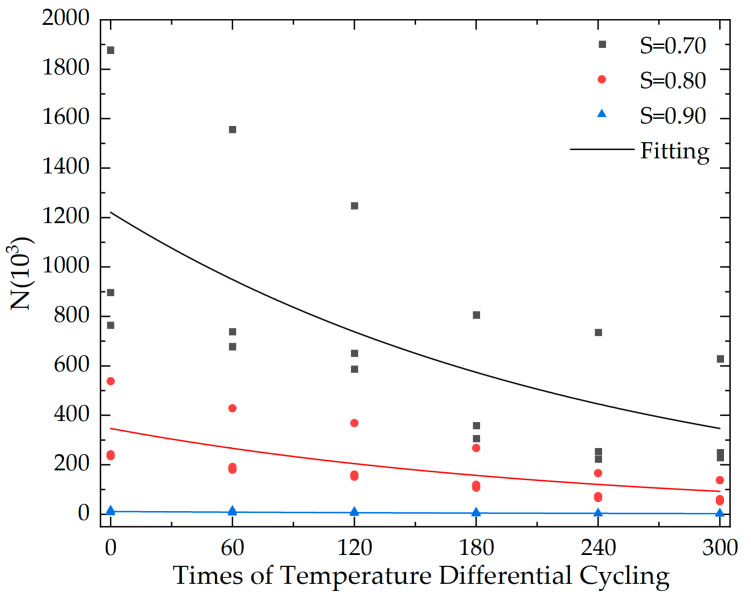
Fatigue life of cement concrete after temperature differential cycling.

The specific values are shown in Table 12, the fitting curve is shown in Figure 9.

To verify the fit of the exponential fitting formula used in this study for compressive fatigue life of concrete, calculations were made using Equation (22) for the fatigue life of concrete under different temperature differential cycling and different stress levels. The calculated fatigue life values were then compared with the experimental values, as shown in Table 13.

It can be found in Table 13 that in 74.7% of the cases, the relative deviation between the calculated value of fatigue life and the experimental value is less than 40%. Therefore, it is considered that Equation (22) can reflect the compressive fatigue life of cement concrete under the effect of temperature differential cycling, providing a reference for practical engineering applications.

Figure 9 reveals that for every incremental increase of 60 cycles in temperature difference, the fatigue life of concrete specimens at the same stress level decreases by approximately 22.23–22.46%; while for the same temperature cycling conditions, an increase in stress level from S_1_ = 0.70 to S_2_ = 0.80, and from S_2_ = 0.80 to S_3_ = 0.90, results in a reduction in the fatigue life of the concrete specimens by 71.50–73.32% and 96.72–96.92%, respectively. Therefore, we believe that both temperature differential cycling and fatigue loading have a significant impact on the compressive fatigue life of cement concrete, with the influence of the stress level being more pronounced. At the same time, it is also noted that, unlike the change in compressive strength of specimens with temperature cycling frequency, the fatigue life of the specimens shows a consistent downward trend. A possible reason is that although the compressive strength of the specimens may increase after 60 cycles of temperature differential cycling, the internal micro-cracks and other defects continue to accumulate under the influence of fatigue loading. At this stage, both the number and width of internal micro-cracks in the specimens are greater than those before the temperature differential cycling, and the speed of propagation of micro-cracks increases with the load during the fatigue loading process, indicating that the overall integrity of the specimens continues to deteriorate, resulting in a reduction in fatigue life.

**Figure 10:**
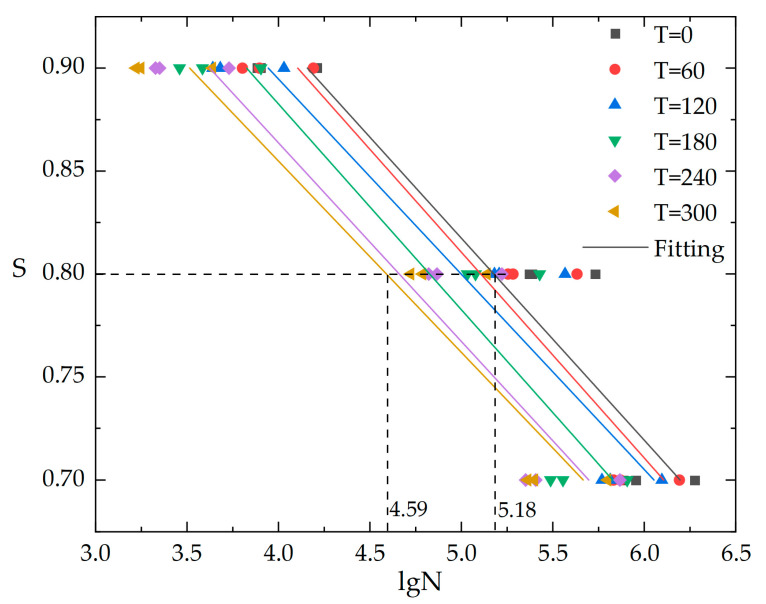
Relationship between S-N of concrete after temperature differential cycling.

For a more intuitive description of the relationship between stress level, temperature differential cycling, and fatigue life of concrete, a linear regression analysis was performed on the fatigue life of cement concrete after different temperature differential cycling. The equation of fatigue life for cement concrete after temperature differential cycling (Equation (23)) was obtained and the S-N curve was plotted (Figure 10).

(23) LgN=kS+b

where k and b are coefficient constants related to the number of temperature cycles, the specific values are as shown in Table 14.

It can be found clearly from Figure 10 that as the number of temperature cycles increases, the S-N curve for cement concrete shifts to the left overall, signifying a significant reduction in fatigue life. At a stress level of S = 0.80, the fatigue life of concrete (lgN) after 300 cycles (T = 300) is even reduced by up to 11.34% compared to T = 0, which confirms the non-negligible impact of temperature cycling on the compressive fatigue life of cement concrete once again. Therefore, to design and predict the fatigue life of cement concrete more scientifically and accurately in actual engineering, it is important to consider not only the environmental stress level but also the effects of temperature differential cycling.

### 3.3. Microstructure of Concrete after Temperature Differential Cycling

The deterioration of compressive strength and the decrease in compressive fatigue life of concrete specimens after temperature differential cycling both, fundamentally, come down to changes in the microstructure. Hence, we approached the issue from the perspective of the microscopic level, employing ultrasonic testing methods to analyze the velocity of sound through concrete specimens after temperature differential cycling statistically.

As is widely known, ultrasonics, as a form of mechanical wave, exhibit different phenomena such as reflection and refraction when passing through various media, which could result in different characteristics such as sound speed and amplitude. While the concrete material, which is a heterogeneous medium, undergoes dissimilar thermal strains in its components under the action of temperature differential cycling. This could lead to internal defects such as micro-cracks and voids in the concrete material, which inevitably affect the propagation path of sound waves, thus manifesting as a loss in wave speed. Therefore, we believe that the change in ultrasonic speed after temperature differential cycling can serve as a statistical measure for characterizing the internal damage patterns of concrete.

**Figure 11:**
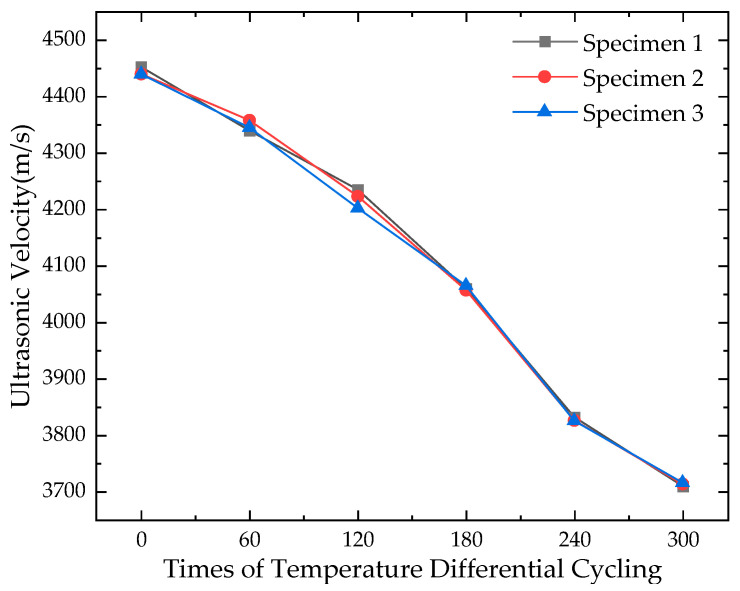
Ultrasonic velocity of specimens after temperature differential cycling.

The results of the ultrasonic test on concrete specimens after different numbers of temperature differential cycling in this study are shown in Table 15, and the curve of the average ultrasonic velocity of three measuring points is drawn as Figure 11.

It can be found from Figure 11 that the ultrasonic velocity of the cement concrete specimen exhibits a gradual decreasing trend with the increase in the number of temperature differential cycles, and the phenomenon of which also indicates, to some extent, that as the temperature differential cycling progresses, damages such as microcracks and pores within the concrete specimen increase, leading to more ultrasonic waves being absorbed, and consequently, a reduction in ultrasonic velocity is observed.

**Figure 12:**
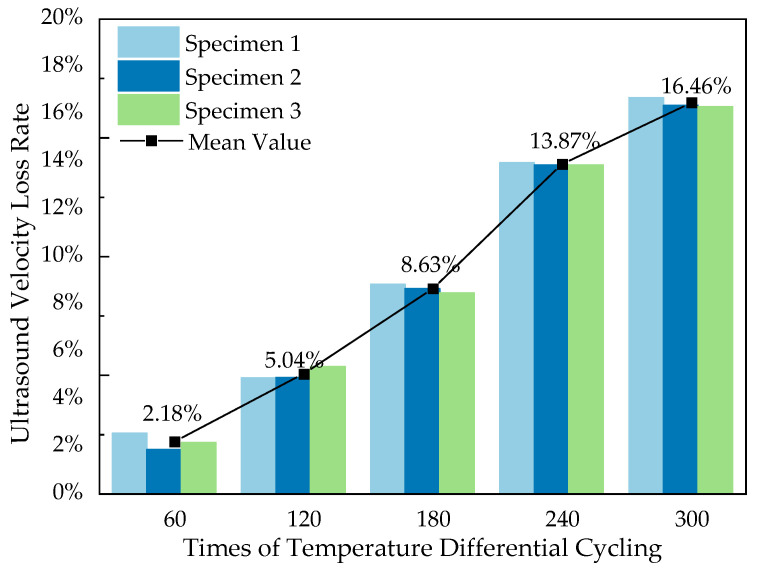
Ultrasound velocity loss of concrete specimens after temperature differential cycling.

The reducing rate of ultrasonic velocity is calculated as shown in Figure 12.

Similar to the trend of strength loss with increasing number of temperature differential cycling, the reducing rate of ultrasonic velocity also shows a trend of gradual increasing. However, it is noted that during the early stages of the temperature differential cycling, there was no negative value in the reducing rate of ultrasonic velocity of the specimen. The possible reason is that in the early process of temperature differential cycling (first 120 cycles), although the hydration of some cement particles increased the hardness of the cement stone, thus improving the strength of the concrete specimen, it could not fully compensate for the microcracks continuously forming and developing at the interface between coarse aggregate and cement paste due to the effect of temperature differential cycling. It is precisely because of the presence of these microcracks that there is a certain degree of loss when ultrasonic waves penetrate the specimen. During the mid-process of temperature differential cycling (120–240 cycles), the cement particles are mostly hydrated, and the effects of temperature differential cycling become the predominant factor, causing a large number of internal microcracks to form in the specimen, which is then reflected in the rapid increase in the reducing rate of ultrasonic velocity. In the later process of the temperature differential cycling (240–300 cycles), the thermal stress between the coarse aggregate and the cement paste is reduced due to the presence of a large number of internal microcracks, resulting in a decreased rate of new microcrack formation. Although the existing cracks continue to absorb energy and gradually expand, the rate of expansion has become stable, so they have no longer a significant impact on the sound velocity, which is also reflected in the gradual leveling off of the rate of increase in the reducing rate of velocity.

**Figure 13:**
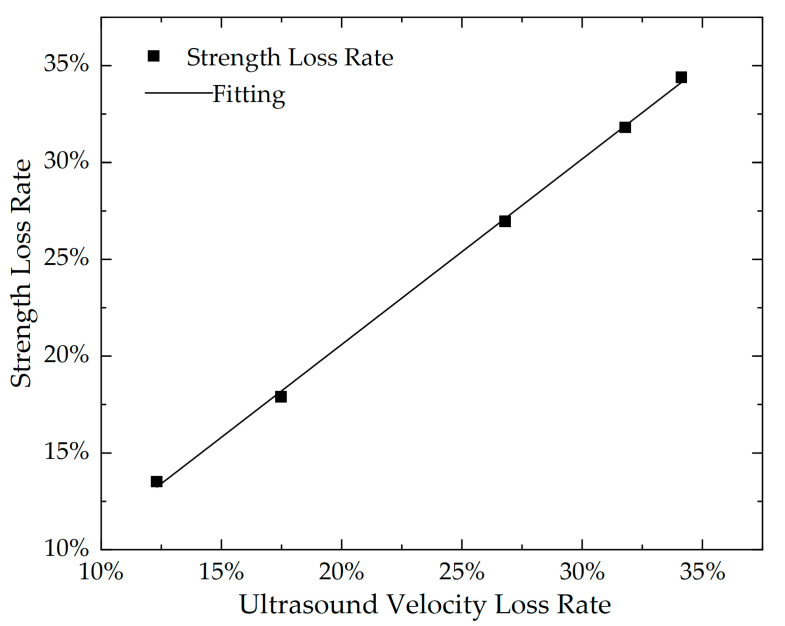
The relationship between ultrasound velocity loss and strength loss.

Considering that the trend in the reducing rate of ultrasonic velocity of the concrete specimen has a strong correlation with the rate of loss in compressive strength after the temperature differential cycling, a linear fitting analysis of the relationship between the strength loss rate (*R*_s_) and the velocity loss rate (*R*_U_) of the concrete specimen was also conducted in this study. The fitting formula is as shown in Equation (24), and the fitting curve is shown in Figure 13.

(24) $$R_{S}=kR_{U}+b$$

where k and b are coefficient constants, which are calculated to be k = 0.47 and b = 0.06, respectively. The degree of fit *R*^2^ reached 0.963, indicating a strong linear relationship between the strength loss rate and the velocity loss rate of the concrete specimen, which also verifies that the ultrasonic velocity loss rate of the concrete specimen can quantitatively characterize the deterioration of concrete strength.

## 4. Conclusions

This research conducted detailed experiments and analysis to explore the impact of temperature differential cycling on the properties of cement concrete, especially its compressive strength and fatigue performance. Based on the background introduction, the literature review, experimental methods, and results analysis, the following main conclusions are drawn:

- The compressive strength of concrete is significantly affected by temperature differential cycling, showing an overall trend of initially increasing and then decreasing. After 60 cycles, the strength of concrete increased by an average of 10.81%. Afterwards, the strength showed a continuous declining trend until after 300 cycles, the average reduction rate reached 19.43%, indicating that temperature differential cycling can cause deterioration of the strength of cement concrete.
- Based on the assumption of log-normal approximation, this study obtained the distribution parameters of the two-parameter Weibull distribution for the fatigue life of cement concrete. Moreover, through the K–S test, it was proven that the distribution of fatigue life of cement concrete after temperature differential cycling conforms well to the two-parameter Weibull distribution.
- By fitting the relationship between concrete fatigue life and the number of temperature differential cycling using an exponential function, it was found that for each increment of temperature differential cycling at the same stress level, the fatigue life is reduced by an average of about 22.35%. Under the same number of temperature differential cycles, for every 0.1 increase in stress level, the fatigue life is reduced by an average of about 84.62%. This indicates that both the effect of temperature differential cycles and the stress level of cyclic loading on the fatigue life of concrete cannot be ignored, and compared to each other, the impact of stress level is more significant.
- Ultrasonic testing was used to investigate the effect of temperature differential cycling on ultrasonic velocity, and it was found that as the number of temperature differential cycling increases, the reducing rate of ultrasonic velocity shows a trend of gradual increase. This phenomenon also confirms that temperature differential cycling can indeed cause the creation and exacerbation of internal damage in concrete.

This study considers the coupled effects of two fatigue factors—thermal fatigue and load fatigue—investigating and discussing the axial compressive strength, axial compressive fatigue life, and ultrasonic wave velocity of concrete under the interaction of these two fatigue factors. This research presents a novel perspective for studying the fatigue performance of concrete. However, it should be noted that this study only discusses one temperature range (20–60 °C) and one type of concrete (C55, cement concrete). In reality, the impact of factors such as temperature ranges, raw materials of concrete, and concrete mix proportions on concrete performance remains a topic worthy of further in-depth investigation.

## Figures and Tables

**Table 1:** Main physical properties of the cement used in the experiment.

Specific Surface Area [m^2^/kg]	Setting Time [min]		Flexural Strength [MPa]		Compressive Strength [MPa]	
	Initial Setting	Final Setting	3d	28d	3d	28d
387	195	242	8.4	8.3	26.5	52.7

**Table 2:** Mix Ratio of Concrete Used in the Experiment (kg/m^3^).

Cement	Fly Ash	Mineral Powder	Medium Sand	Crushed Stone	Water	Pumping Agent
345	50	70	698	1035	165	14.5

**Table 3:** Quantity of Specimens Used in the Experiment.

Experiment	Size of Specimen [mm]	Number of Specimens [Pieces]
axial compression strength test	100 × 100 × 300	18
axial compression fatigue test	100 × 100 × 300	54
ultrasonic test (reference specimen)	100 × 100 × 300	3

**Table 4:** Axial compressive strength of the concrete specimens after temperature differential cycling.

Ti	Failure Load (kN)				Compressive Strength (MPa)			
	Specimen Ti-1	Specimen Ti-2	Specimen Ti-3	Standard Deviation	Specimen Ti-1	Specimen Ti-2	Specimen Ti-3	Standard Deviation
0	509.2	463.8	422.1	43.56	48.37	44.06	40.10	4.14
60	549.9	532.8	463.2	45.92	52.24	50.62	44.00	4.36
120	467.3	518.7	438.7	40.54	44.39	49.28	41.68	3.85
180	387.5	464.6	421.4	38.64	36.81	44.14	40.03	3.67
240	427.7	376.4	376.6	29.56	40.63	35.76	35.78	2.81
300	343.8	410.9	369.4	33.86	32.66	39.04	35.09	3.22

where Ti represents the times of temperature differential cycling, i = 0, 60, 120, 180, 240, 300.

**Table 5:** Slope and intercept of the curve “Compressive Strength—Number of temperature differential cycling”.

T	Slope	Intercept
0–60	0.080	44.18
60–120	−0.064	52.79
120–180	−0.080	54.69
180–240	−0.049	49.14
240–300	−0.030	44.56

**Table 6:** Compressive strength loss of concrete specimens after temperature differential cycling.

Ti	Strength Loss Rate		
	Specimen Ti-1	Specimen Ti-2	Specimen Ti-3
60	−7.99%	−14.88%	−9.74%
120	8.23%	−11.84%	−3.93%
180	23.90%	−0.17%	0.17%
240	16.01%	18.84%	10.78%
300	32.48%	11.41%	12.49%

where Ti represents the times of temperature differential cycling, i = 0, 60, 120, 180, 240, 300.

**Table 7:** Compressive fatigue life of concrete specimens after temperature differential cycling.

T	Fatigue Life (N)		
	S = 0.70	S = 0.80	S = 0.90
0	765,849	235,183	7647
	897,656	242,440	8015
	1,876,975	538,494	16,202
60	678,593	180,078	6350
	739,652	191,529	7881
	1,556,487	428,596	15,517
120	587,636	151,773	4807
	652,316	160,906	4380
	1,248,567	369,175	10,721
180	307,685	107,872	2877
	359,647	119,389	3841
	806,943	268,095	8025
240	224,682	66,299	2128
	255,298	73,549	2240
	736,347	166,666	5362
300	230,248	52,684	1660
	250,264	61,289	1763
	629,534	138,007	4348

**Table 8:** Correspondence of ω(*β*)–*β*.

*β*	*ω*(*β*)	*β*	*ω*(*β*)	*β*	*ω*(*β*)	*β*	*ω*(*β*)	*β*	*ω*(*β*)	*β*	*ω*(*β*)
1.000	1.000	1.375	1.359	1.750	1.696	2.125	2.020	2.500	2.337	2.875	2.648
1.025	1.025	1.400	1.382	1.775	1.718	2.150	2.042	2.525	2.358	2.900	2.669
1.050	1.050	1.425	1.405	1.800	1.740	2.175	2.063	2.550	2.379	2.925	2.690
1.075	1.074	1.450	1.427	1.825	1.761	2.200	2.084	2.575	2.400	2.950	2.710
1.100	1.099	1.475	1.450	1.850	1.783	2.225	2.105	2.600	2.420	2.975	2.731
1.125	1.123	1.500	1.473	1.875	1.805	2.250	2.126	2.625	2.441	3.000	2.751
1.150	1.147	1.525	1.495	1.900	1.827	2.275	2.148	2.650	2.462	3.025	2.772
1.175	1.171	1.550	1.518	1.925	1.848	2.300	2.169	2.675	2.483	3.050	2.793
1.200	1.195	1.575	1.540	1.950	1.870	2.325	2.190	2.700	2.504	3.075	2.813
1.225	1.219	1.600	1.563	1.975	1.892	2.350	2.211	2.725	2.524	3.100	2.834
1.250	1.242	1.625	1.585	2.000	1.913	2.375	2.232	2.750	2.545	3.125	2.854

**Table 9:** Calculated values of Weibull distribution parameters for fatigue life of concrete.

T	*β*			*η*		
	S = 0.70	S = 0.80	S = 0.90	S = 0.70	S = 0.80	S = 0.90
0	2.65153	2.70998	3.09148	1,322,054	378,949	11,835
60	2.80484	2.61542	2.73474	1,108,626	298,681	11,107
120	3.20129	2.53063	2.56191	923,239	254,643	7435
180	2.41022	2.52001	2.34681	551,245	185,047	5524
240	1.81007	2.48367	2.39973	451,268	114,525	3636
300	2.19658	2.40838	2.29045	414,697	94,214	2904

**Table 10:** K–S test for distribution of fatigue life of cement concrete specimens at S = 0.70, T = 0.

Serial Number	*x*_i_	*F*_n_(*x*)	*P*_n_(*x*)	*D_i_*	*D*_max_	*D*_C_
1	765,849	0.3333	0.2095	0.1238	0.3656	0.7340
2	897,656	0.6667	0.3011	0.3656		
3	1,876,975	1.0000	0.9206	0.0794		

**Table 11:** K-S tests of fatigue life distribution of concrete specimens after different stress levels and temperature differential cycling.

S	*D*_max_					
	T = 0	T = 60	T = 120	T = 180	T = 240	T = 300
0.70	0.3656	0.3918	0.3864	0.3663	0.3667	0.3858
0.80	0.4089	0.3981	0.3979	0.3846	0.3835	0.3678
0.90	0.4077	0.3429	0.4394	0.3197	0.3981	0.3937

**Table 12:** Coefficient constants of fitting equation.

S	a	b	c
S = 0.70	1,221,042.10	−0.00419	0
S = 0.80	1	−0.00440	12.75703
S = 0.90	11,387.77	−0.00461	0

**Table 13:** Calculated values of fatigue life and relative deviation.

T	S = 0.70		S = 0.80		S = 0.90	
	Calculated Value	Relative Deviation	Calculated Value	Relative Deviation	Calculated Value	Relative Deviation
0	1,221,042	37.28%	346,983	32.22%	11,388	32.85%
		26.48%		30.13%		29.62%
		−34.95%		−35.56%		−29.72%
60	949,618	28.54%	266,473	32.42%	8636	26.47%
		22.11%		28.12%		8.75%
		−38.99%		−37.83%		−44.34%
120	738,529	20.43%	204,645	25.84%	6549	26.60%
		11.67%		21.37%		33.12%
		−40.85%		−44.57%		−38.91%
180	574,362	46.43%	157,162	31.36%	4967	42.07%
		37.38%		24.03%		22.67%
		−40.49%		−41.38%		−38.11%
240	446,688	49.70%	120,696	45.07%	3766	43.49%
		42.85%		39.06%		40.53%
		−39.34%		−27.58%		−29.76%
300	347,394	33.72%	92,691	43.16%	2856	41.90%
		27.96%		33.88%		38.29%
		−44.82%		−32.84%		−34.31%

**Table 14:** Coefficient constants of fitting equation.

T	k	b	*R* ^2^
0	−10.1896	13.3289	0.90
60	−10.0044	13.1094	0.91
120	−10.5440	13.4339	0.90
180	−10.0050	12.8303	0.87
240	−10.3635	12.9511	0.89
300	−10.7586	13.1972	0.91

**Table 15:** Ultrasonic velocity of specimens after temperature differential cycling (m/s).

T	Specimen	Measuring Point 1	Measuring Point 2	Measuring Point 3	Mean Value
0	1	4435	4467	4456	4453
	2	4456	4428	4437	4440
	3	4448	4439	4433	4440
60	1	4354	4327	4338	4340
	2	4366	4343	4365	4358
	3	4332	4336	4367	4345
120	1	4236	4235	4235	4235
	2	4221	4226	4223	4223
	3	4196	4189	4222	4202
180	1	4067	4060	4053	4060
	2	4052	4053	4066	4057
	3	4055	4073	4067	4065
240	1	3833	3822	3841	3832
	2	3821	3835	3822	3826
	3	3818	3830	3828	3825
300	1	3715	3712	3702	3710
	2	3718	3709	3713	3713
	3	3716	3719	3712	3716

## Data Availability

The data used to support the findings of this study are available from the authors upon request.
